# The COMBAT Project: study protocol for the development of a core outcome set for morbidity following surgery in paediatric brain tumour patients

**DOI:** 10.1186/s13063-025-09004-4

**Published:** 2025-08-11

**Authors:** Sandhya Trichinopoly Krishna, Nicola Harman, Conor Mallucci, Barry Pizer, Michael D. Jenkinson, Kristian Aquilina, Kim Bull, Jon Foss-Skiftesvik, Helen Hartley, James Hayden, Colin Kennedy, Ulrich W. Thomale, Sophie Wilne, Jeffrey H. Wisoff, Faris Bouaouiche, Liz Hull, John Robinson, Hannah L. Vickers, Carrol Gamble

**Affiliations:** 1 Department of Neurosurgery, Alder Hey Children’s NHS Foundation Trust, Liverpool, UK; 2 University of Liverpool, Liverpool, UK; 3 Department of Neurosurgery, The Walton Centre NHS Foundation Trust, Liverpool, UK; 4 Department of Health Data Science, University of Liverpool, Liverpool, UK; 5 Paediatric Oncology Department, Alder Hey Children’s NHS Foundation Trust, Liverpool, UK; 6 Institute of Systems, Molecular and Integrative Biology, University of Liverpool, Liverpool, UK; 7 Department of Neurosurgery, Great Ormond Street Hospital for Children NHS Foundation Trust, London, UK; 8 Faculty of Medicine, University of Southampton, Southampton, UK; 9 Department of Neurosurgery, Copenhagen University Hospital Rigshospitalet, Copenhagen, Denmark; 10 Department of Physiotherapy, Alder Hey Children’s NHS Foundation Trust, Liverpool, UK; 11 Department of Pediatric Neurosurgery, Charité – Universitätsmedizin, Berlin, Germany; 12 Department of Paediatric Oncology, Nottingham University Hospitals NHS Trust, Nottingham, UK; 13 Division of Pediatric Neurosurgery, NYU Langone Health, Hassenfeld Children’s Hospital, Nottingham, New York USA; 14 Alder Hey Children’s NHS Foundation Trust, Liverpool, UK; 15 Thumbs Up For Charlie, Preston, UK; 16 Liverpool Clinical Trials Centre, University of Liverpool, Liverpool, UK

**Keywords:** Paediatric, Child, Central nervous system, Brain tumour, Neuro-oncology, Surgery, Neurosurgery, Morbidity, Adverse outcome, Core outcome set

## Abstract

**Background:**

Central nervous system tumours affecting the brain and spine are the most common solid tumour site in the paediatric population and the most common causes of cancer death in children and young people. They are associated with high morbidity both from the tumour and the interventions used to treat them. Postoperative morbidity reporting following surgery for paediatric brain tumours is poor. This is due to variability of outcomes measured and reported and the lack of a common language when reporting adverse outcomes. One solution is to develop a core outcome set which will stipulate the minimum postoperative outcomes that should be reported. The COMBAT (Core Postoperative Morbidity Set for Paediatric Brain Tumours) Project will develop a core set of adverse outcomes that can be applied to paediatric brain tumour patients undergoing surgery.

**Methods and analysis:**

This protocol has been developed using the COS-STAD (Core Outcome Set-Standards for Development) recommendations and the COS-STAP (Core Outcome Set-STAndardised Protocol Items) statement. A systematic review will identify adverse outcomes reported in the literature and how they are measured. Outcomes of importance to patients and their carers will be identified from semi-structured qualitative interviews with patients and their carers from Alder Hey Children’s Hospital, Liverpool, UK. Consensus on the most important harms will be sought using a two-round eDelphi survey completed by national and international participants including health professionals, researchers, patients and their carers. Results of the eDelphi survey will be assessed against a pre-defined definition of consensus and discussed at an international consensus meeting attended by participants of the eDelphi survey.

**Discussion:**

There is a clear need for a common language to harmonise measurement and reporting of morbidity following surgery for paediatric brain tumour patients. This project will define postoperative adverse outcomes that are of critical importance to key stakeholders. It will standardise surgical morbidity outcome measurement and reporting in both research studies and routine clinical practice, enabling comparison across different trials, studies and clinical services. It will lay the groundwork for future research in paediatric brain tumour surgical morbidity.

**Study registration:**

This study is registered with the COMET database as Study 1968 (https://www.comet-initiative.org/Studies/Details/1968), registration date: 26/10/2021.

**Supplementary Information:**

The online version contains supplementary material available at 10.1186/s13063-025-09004-4.

## Background

Over 400 children are diagnosed with a central nervous system (CNS) tumour per year in the UK. CNS tumours cause 35% of cancer deaths in childhood which equates to over 85 children a year in the UK. [1] Globally, incidence of new paediatric CNS tumours in 2019 was 47,600, with 23,500 deaths due to these tumours in the year. [2] Survival rates at 5 years are > 75%, with most survivors living for several decades. As a result, prevalence rates increase with age, with an estimate of 1 in 4000 adults being a survivor of a brain tumour in childhood [3, 4].

Although novel targeted treatments for selected tumour types are emerging, surgery remains the first-line treatment for most tumours. The extent of surgical resection is a key determinant of prognosis. [5] Paediatric brain tumour surgery is associated with substantial postoperative morbidity due to challenges related to tumour access, the surgical corridor, tumour biology and proximity to eloquent neurovascular structures. Postoperative morbidity is frequent, with studies reporting occurrence in about two-thirds of all patients. [6, 7] Complication rates following surgery range between 10 and 50% which can lead to long-term disability [8].

Reporting postoperative morbidity associated with paediatric brain tumours is challenging due to their pathological heterogeneity and location specific symptoms and morbidity. The pathology and anatomical location dictate the surgical aggressiveness needed to achieve adequate disease control, which may result in postoperative morbidity. In addition, adjuvant therapies may compound overall tumour-associated morbidity.

Transparent and reproducible morbidity reporting provides a standardised way to compare adverse outcomes in clinical or research studies and provides a benchmark to compare clinical services. The lack of consistency in outcomes measured and reported in paediatric neuro-oncology makes establishing a benchmark for comparison of surgical interventions difficult. [9] A range of morbidity tools used to retrospectively grade postoperative outcomes have been shown to be inadequate [6, 10] due to consistently underestimating the clinical importance of adverse outcomes following surgery. In addition, the use of patient-reported outcome measures (PROMs) in paediatric neuro-oncology outpatient clinics is not yet standard [11] with ongoing research and discussions with patient groups to determine the most appropriate and relevant PROM for use. [12, 13] There is currently a shift away from the biomedical model of disability towards a more social model of disability, which highlights the importance of quality of survival (QoS) measures and a multidisciplinary approach to care. Research has highlighted the challenges associated with the standardised implementation of outcome assessments in children across clinical trials in Europe as a whole, due to language barriers and availability of the same assessments in each country. [3, 4]

There is a clear need identified by the scientific community to standardise postoperative morbidity reporting in paediatric neuro-oncology surgery. The Get It Right First Time (GIRFT) Programme National Speciality Report for Paediatric Neurosurgery states there is no standard to collect or publish outcome data in paediatric neuro-oncology, with no clinical audits to benchmark surgical units. A key recommendation of this national report is ‘the development of a robust, validated and evidence based reporting system for outcomes in paediatric brain tumour surgery’ with calls for a national working group to be set up urgently to ‘agree a set of core outcome measures’. [14] Scientific committees such as the European Society for Paediatric Oncology (SIOP-E) and the British Paediatric Neurosurgical Group (BPNG) have called for standardisation of postoperative morbidity reporting in paediatric neuro-oncology surgery, and agreement of a ‘core plus’ approach for QoS measures, in which core assessments are recommended for clinical trials. [3, 4]

One way to do this is the development of a core outcome set (COS). This is a set of outcomes that should be reported as a minimum in trials or studies of a particular health condition. The COMBAT Project aims to seek consensus on which postoperative adverse outcomes are most important to key stakeholders, including patients and carers. The project will also collate information on how these are measured in current literature to inform future research into measurement of morbidity and standardising the tools used to reported the adverse outcomes included in the final COS. [15]

### Rational for core outcome set

A well developed and disseminated COS can improve the quality of research and reduce research waste. They are applied in other medical and surgical specialities with good uptake and effect. A notable example of development and uptake of the use of COS in a specialty is OMERACT (Outcome Measures in Rheumatology), which over the last 20 years has standardised outcome reporting in rheumatology trials whilst still ensuring a patient centred approach. The uptake of the rheumatoid arthritis COS has continued to increase over time since its publication in 1994 with reporting of the complete COS in 81% of trials and publications. [16] Most COS are disease based [17–19] but they can also be applied to groups of disease pathologies such as multiple cancer types in adults [18] and have been successfully developed in adult surgical neuro-oncology. [20, 21] There is no COS, published or in development, for paediatric neuro-oncology surgery or evaluating postoperative morbidity in neurosurgery. Development of a COS is an effective way to address our aims whilst maximising key stakeholder group involvement.

CNS tumours in children constitute a relatively rare and heterogenous group, with over 100 individual tumour types. [22] This means a tumour subtype specific COS, although useful, may not have as much relevance or uptake as a broader COS. Postoperative complications of paediatric brain tumours can be related to common factors such as anatomical location and approach used in surgery rather than specific pathology, and so a broader COS is more relevant for key stakeholders. This project will draw upon methodological aspects from a range of different types of COS and is anticipated to require a novel approach to these methodologies to accommodate the inclusion of children.

## Methods

### Project scope

The final COS will be applied to research trials, studies and clinical practice. It includes paediatric patients (children and young people) with a brain tumour up to and including the age of 18 years at the time of diagnosis. All paediatric neuro-oncology surgical operations including surgical resection and biopsy will be included in this project. The definition of neuro-oncology pathology in this project includes both benign and malignant tumour types. The scope of the COS has been defined as per the Core Outcome Set-Standards for Development (COS-STAD) recommendations [23] and can be seen in Table 1. This protocol has been developed in line with the Core Outcome Set-STAndardised Protocol Items (COS-STAP) statement (see Additional file 1). [24]

**Table 1:** Core Outcome Set-Standards for Development recommendations (COS-STAD) [23] as applied to The COMBAT Project

Domain	Standard	Methodology	The COMBAT Project
Scope specification	1	The research or practice setting(s) in which the COS is to be applied	Clinical practice and research about surgical interventions and postoperative morbidity in paediatric neuro-oncology patients
	2	The health condition(s) covered by the COS	All paediatric intracranial brain tumours
	3	The population(s) covered by the COS	Children and young adults aged 18 years or below at time of diagnosis for their brain tumour
	4	The intervention(s) covered by the COS	Definitive surgical interventions—biopsy, subtotal, partial and gross total resection, and cyst drainage
Stakeholders involved	5	Those who will use the COS in research/clinical practice	Healthcare workers and researchers involved in the care of postoperative paediatric neuro-oncology patients Healthcare roles included will be: Medical staff Nursing staff Allied healthcare professionals
	6	Healthcare professionals with experience of patients with the condition	Healthcare professionals as outlined above from multiple subspecialties with involvement in the delivery of surgical interventions and care of postoperative patients with brain tumours
	7	Patients with the condition or their representatives	Paediatric patients who received a diagnosis of a brain tumour up to and including the age of 18 will be included, along with relatives and carers of such patients
Consensus process	8	The initial list of outcomes considered both healthcare professionals’ and patients’ views	1) A systematic literature review of previously published and current ongoing trials and clinical studies and patient-reported outcome measures reporting postoperative morbidity in paediatric neuro-oncology patients 2) Qualitative interviews with patients and carers to determine outcomes of importance to them
	9	Scoring process and consensus definition	1) 1–9 Likert style scale (1 not that important, 9 critically important) 2) Alternative scoring system such as modified Wong-Baker scale [25] or a traffic light system [26] which will correlate to the Likert style scale for younger children to use 3) Consensus definition—outcomes will need to be rated as critically important (ratings of 7–9) by 80% of stakeholders within each stakeholder group and fewer than 15% scoring the outcome as not important (ratings of 1–3)
	10	Criteria for including/dropping/adding outcomes	• Participants of the eDelphi survey will be able to recommend outcomes for inclusion after the first round for inclusion in the second round • Recommendations of new outcomes for round two will be discussed with the Study Advisory Group prior to inclusion • All outcomes that are included in round one will be rated in round two
	11	Care was taken to avoid ambiguity of language used in the list of outcomes	• Both clinical and plain language definitions informed by the qualitative interviews will be included as approved by the Study Advisory Group • All materials will be reviewed by the Study Advisory Group including patient and carer research partners and pilot tested with patients and healthcare professionals. A think aloud process will be employed at this stage to check understanding

### Aims and objectives

The aims of this project are to:

1. Develop a core outcome set for postoperative adverse outcomes following surgery in children with brain tumours.
2. Collate information on how the outcomes in the core outcome set have been measured and reported to inform future research into how, when and by whom each outcome should be optimally measured. Determination of how each outcome should be measured is outside the scope of this project and will be determined in future research.

The objectives of this project are:

- Formation of a Study Advisory Group and development of a detailed study protocol for peer review in line with the COS-STAP guidelines (Core Outcome Set-STAndardised Protocol Items). [24]
- Early engagement of key stakeholders internationally, including healthcare professionals, researchers, patients and carers.
- Identify adverse outcomes and how they are measured following paediatric brain tumour surgery through systematic literature reviews of published and current ongoing clinical trials, clinical studies and patient-reported outcome measures in the literature.
- Carry out semi-structured qualitative patient interviews to identify any outcomes not identified from the systematic review.
- Collate outcomes identified from the systematic review and patient and carer interviews to identify a comprehensive list of outcomes.
- Seek consensus on the most important adverse outcomes using an eDelphi survey with three key stakeholder groups—(1) patients and carers, (2) healthcare professionals and (3) researchers.
- Conduct an international consensus meeting to ratify the COS.
- Publication, presentation and dissemination of the final COS in open access journals.

### Definition of an ‘adverse outcome’

An adverse outcome is defined as an event resulting in a negative impact. [27] Adverse outcomes are defined in this study as outcomes following paediatric brain tumour surgery that cause a negative effect on the patient. They may be temporary or permanent, and for the purposes of this study may or may not require further intervention. All types of adverse outcomes will be recorded and included. This includes but is not limited to physical, neurological, psychological, emotional, cognitive and functional adverse outcomes. Adverse outcomes will be classified as outlined by COMET taxonomy into domains. [15] The definition of adverse outcome will encompass the terms ‘morbidity’ and ‘harms’ that may be encountered in our systematic review. Adverse outcomes will be referred to as ‘unwanted outcomes’ in the patient/public documentation and involvement, as discussed with the patient and parent research partners in the Study Advisory Group.

### Study oversight

#### Study management group (SMG)

The Study Management Group (SMG) is responsible for overseeing day to day development of this project. It includes experts in clinical trials, research methodology and paediatric oncology and neurosurgery based at the University of Liverpool, Alder Hey Children’s Hospital and The Walton Centre in Liverpool, UK.

#### Study advisory group (SAG)

An international Study Advisory Group (SAG) will provide study oversight. Members include healthcare professionals, researchers, patients and carers. Patients and carers were recruited from Alder Hey Children’s Hospital and healthcare workers and researchers were recruited at international conferences and scientific meetings.

### Other collaborations

The European Society for Paediatric Oncology (SIOP-E), the Society of British Neurological Surgeons (SBNS) and the British Paediatric Neurosurgical Group (BPNG) are supporting this initiative. The Brain Tumour Charity [28] and Thumbs Up For Charlie Charity [29] will provide support in patient and carer recruitment. This project will be completed in and supported by the Trials Methodology Research Partnership Outcomes working group and COS sub-group, an international group of researchers and public research partners with expertise and interest in COS development and uptake.

### Patient and public involvement

Patients and carers will be involved at all stages of the project. Patient and public involvement in COS development has been shown to reduce attrition during the consensus process. [30] Two patients (who have previously had surgery for a brain tumour—FB, HLV) and two carers (LH, JR) are members of the SAG as research partners to ensure consideration of patient/parent perspectives throughout the whole process. They will help guide the project, including development of the protocol and review of progress and decision-making at key stages of the project. Their input will be sought on all materials for patient/carer participants, including advertising materials, interview topic guides, outcome definitions, eDelphi structure and the consensus meeting plan.

### Ethics and data protection

Ethical approval for all stages of this project has been received from the West Midland—South Birmingham Research Ethics Committee (REC reference 24/WM/0219) and the HRA and HCRW (IRAS project ID: 326,584).

The main ethical considerations for this project will arise from the involvement of paediatric patients and carers. A distress protocol with an information sheet signposting organisations for further support has been developed to support participants. Informed consent and assent will be sought prior to participation, and all data collected will be pseudonymised.

Storage of data and data handling will be in accordance with the Alder Hey Children’s Hospital and University of Liverpool local data protection guidelines and the Data Protection Act 1998 and 2018. Identifiable data for patients identified from Alder Hey Children’s Hospital, both through outpatient clinics and local databases, will be stored on the K drive at Alder Hey Children’s Hospital in password protected files. Only pseudonymised data will be transferred out of the Trust in line with Alder Hey Children’s Hospital policy to minimise risks associated with data transfer. Data will be stored in Microsoft Excel spreadsheets, [31] NVivo [32] and Delphi management software, all of which will be password protected, encrypted and stored on the University of Liverpool’s secure servers. This is in line with Alder Hey Children’s Hospital data protection guidelines. All identifiable data will be destroyed at the end of the project, with only anonymised information stored for 10 years (or longer if of historical significance) on the University of Liverpool Open Repository in line with University of Liverpool and Alder Hey Children’s Hospital guidelines.

A study website (www.thecombatproject.org) has been developed to share study information including videos explaining the project to support recruitment of paediatric patients as a key demographic. The use of more interactive resources such as animated videos to explain what involvement in a COS entails has been shown to encourage participation and understanding in the consent process in children and adolescents. [17, 33–35] There is a link to an expression of interest form but no participant data will be stored on the website.

## Work packages

**Fig. 1:**
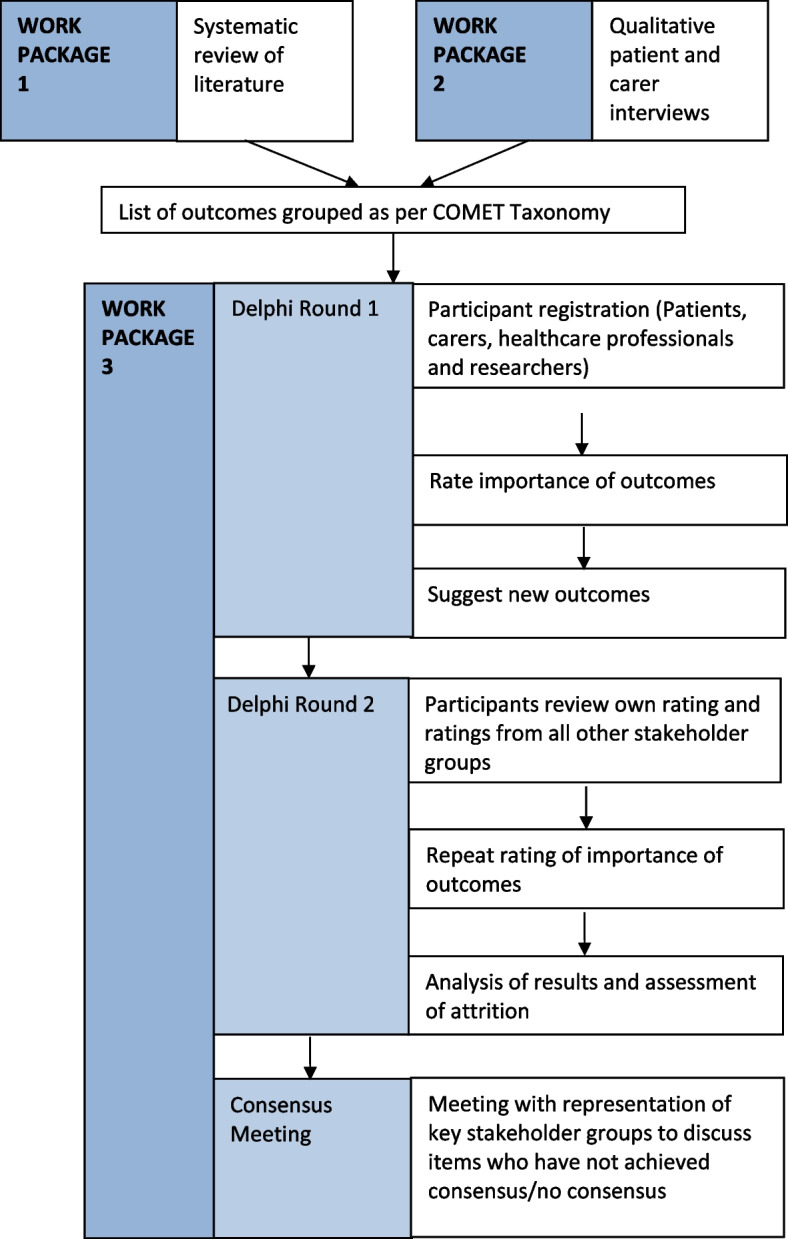
Key stages of The COMBAT Project divided into 3 work packages

We will achieve our aims through three linked work packages (WP) (Fig. 1):

- Work package 1 (WP1)—systematic review to identify adverse outcomes reported in the literature.
- Work package 2 (WP2)—qualitative interviews to generate a list of adverse outcomes important to patients and carers.
- Work package 3 (WP3)—consensus process—eDelphi survey and consensus meeting.

### WP1—Systematic review to identify outcomes reported in the literature

A systematic review will be conducted to identify adverse outcomes reported in the literature. These outcomes will be combined with those from the qualitative interviews in WP2 to create a comprehensive eDelphi survey. This systematic review has been registered on the PROPSERO website (registration number CRD42024504042).

#### Research question

What adverse outcomes are measured and reported in ongoing and published clinical studies, trials and patient-reported outcome reviews assessing the effectiveness and morbidity of surgical interventions in paediatric neuro-oncology patients?

#### Types of participants

The population covered includes paediatric neuro-oncology patients (children and young people) with a brain tumour up to and including the age of 18 years at the time of diagnosis, of either sex, and who have undergone a neurosurgical intervention. Diagnosis of an intracranial brain tumour will be radiologically and histologically confirmed. Patients with all subtypes of benign and malignant intracranial brain tumour will be included.

#### Types of interventions

Paediatric patients who have undergone any cranial neurosurgical intervention will be eligible for this study. This includes definitive operative treatments such as resection or biopsy or cyst drainage (endoscopic, open or stereotactic/burr hole). Studies describing adjunct surgical interventions, isolated non-surgical interventions and non-intracranial tumours will be excluded. Studies describing a combination of non-surgical and surgical interventions will be included if separate outcomes for surgical treatment can be identified. If it is not possible to differentiate between outcomes affecting adult or paediatric patients, then the study will not be included. The full inclusion and exclusion criteria can be found in Table 2.

**Table 2:** Inclusion and exclusion criteria for systematic review

Inclusion criteria	Exclusion criteria
• Studies on paediatric patient up to and including the age of 18 at the time of diagnosis • English language studies (or studies published in another language but with validated English translation) • All retrospective and current ongoing studies, trials and patient-reported outcome measures • Full text • All brain tumours—benign and malignant • Surgical interventions—resection and biopsy • Clinical studies published in the last 10 years • Clinical studies from all years suggested by SAG members • Ongoing clinical trials identified from clincialtrials.gov	• Adult patients over the age of 18 at diagnosis • Spinal tumours • Studies including non-neuro-oncology pathologies • Adjuvant surgical interventions such as external ventricular drains, endoscopic third ventriculostomy or ventriculoperitoneal shunts • Isolated non-surgical interventions such as such as pharmacological therapy, chemotherapy, radiotherapy, radiosurgery, peri operative and supportive treatments or therapies • Vascular lesions and non-intracranial tumours such as bony skull lesions and extracranial schwannoma lesions • Resections for epilepsy caused by non-neoplastic lesions such as focal cortical dysplasia • Studies looking only at management of postoperative deficits such as cerebellar mutism with no definitive post-surgical outcomes outlined • Studies published in another language apart from English and with no validated translation into English • Abstract only publications • Clinical studies older than 10 years old (unless suggested by SAG members) • Studies with no outcome measures, e.g. technical tips

#### Types of study

Studies for inclusion in the systematic review will be identified from 6 key sources:

1. Published clinical studies

Relevant literature will be identified from electronic databases including PubMed, Cochrane Library, EMBASE, CINAHL, AMED, MEDLINE and NHS knowledge and library hub. Published observational studies, case studies, case series, clinical studies and published clinical trials will be included. If there are multiple papers relating to the same study, then data will be extracted from each paper to ensure that all reported outcomes are recorded. Systematic reviews and meta-analysis studies will not be included but each paper referenced will be extracted and checked for adherence to the inclusion criteria.

Studies from the last 10 years will be included and outcomes will be extracted until data saturation is attained. Assessment for data saturation will take place after each 20 studies, defined as no new outcomes identified when compared to outcomes extracted from the previous 20 papers. If this is not achieved, then we will include references from the included papers and extend our search further back in 10-year increments.

(2) Ongoing clinical trials

Clinical trial registries including ClinicalTrials.gov, ISRCTN and the International Clinical Trials Registry Platform will be searched to identify relevant trials for inclusion in the systematic review.

Clinical trials from all years will be included due to small number of clinical trials in surgical paediatric neuro-oncology. Data will be identified and recorded in the same way as clinical studies.

(3) Patient-reported outcome measures (PROMs)

Paediatric neuro-oncology has not fully integrated PROMs into common practice, reflected in the high variability of tools used. Studies that use PROMs to assess adverse outcomes reported by patients, or their families, will be included if they have been exclusively applied to paediatric postoperative neuro-oncology patients. The impaired domain within the PROM, and the adverse outcome it reflects, will be recorded, rather than the PROM itself. The PROM used to assess the adverse outcome reported will be recorded separately. This will be used in future research when assessing how to measure each outcome included in this COS. Data extraction and analysis will be assessed as per COMET (Core Outcome Measures in Effectiveness Trials) recommendation. [15, 36]

(4) Reference from ‘Reporting morbidity associated with pediatric brain tumor surgery: are the available scoring systems sufficient?’. [10]

This study was conducted at Alder Hey Children’s Hospital and identified that current ways of measuring morbidity following surgery in paediatric brain tumour patients were inadequate, leading to the development of this project. A comprehensive literature review was conducted at the time of publication by the authors. To ensure that we are capturing all papers of relevance, we will include references from this paper.

(5) Suggestions from members of the SAG

Papers suggested by members of the SAG will be reviewed to confirm if they have been identified in the search strategy. Papers not identified from the searches will be included in the systematic review even if they fall outside the time limit in the inclusion criteria. The studies will otherwise be subject to the same inclusion and exclusion criteria as those identified from the searches of electronic databases.

(6) Surgical trials identified from qualitative synthesis

A qualitative synthesis of outcomes reported by patients who have undergone surgery for a brain tumour and their families will be completed. Any papers identified with surgical outcome will be included in the systematic review.

#### Data collection

Studies and trials identified will be downloaded and uploaded onto the online platform Rayyan. [37] Following deduplication, each study’s title and abstract will be screened to ensure it meets the appropriate inclusion and exclusion criteria. If there is any uncertainty, the whole paper will be screened. Screening will be conducted by the lead investigator (STK) with batch check verification from a second author. Any uncertainty about the inclusion of any of the studies will be discussed at the SMG.

#### Data extraction

Data from the eligible studies will be extracted into a Microsoft Excel [31] worksheet by the lead investigator (STK). Outcomes from a pilot of 5 studies generated randomly by Rayyan [37] will be extracted first and checked with a second author (NH) to ensure consistency. All outcomes, both favourable and unwanted will be extracted for purposes of transparency.

Outcomes will then be extracted from the identified studies in chronological order, starting from the most recent. Assessment for data saturation will take place after each 20 studies, defined as no new outcomes identified when compared to outcomes extracted from the previous 20 papers. All studies suggested by the SAG will be included in addition to the 20 most recent studies to ensure seminal papers in the field are captured. Data extraction will conclude after data saturation has been achieved.

Data about the type of study as well as the adverse outcomes reported will be extracted. The study type, geographical location, date of publication, first author, DOI, journal of publication and title will be recorded. The type of tumour, surgical intervention, length of follow-up and the outcomes reported in verbatim will be recorded. Measurement methods/instruments used to report these outcomes will be recorded as well as the time points or length of times over which the outcomes were recorded. If a study includes both adult and paediatric patients, then the outcomes specifically related to the paediatric cases will be extracted. If the same outcome has been recorded at multiple time points, it will just be recorded once. If the same outcome has been reported for different tumour types or surgical interventions, then the outcome will be recorded for each of these to enable comparison between different subgroups. If different scales or measurements are used to record the same outcome, then both will be recorded. Outcomes will be classified as favourable and unwanted. Favourable outcomes will reflect treatment goals whilst unwanted outcomes will be consistent with adverse outcomes. Any equipoise about classification of the outcomes will be discussed in the SMG and presented to the SAG.

#### Data analysis

Analysis of the data extracted will be conducted in Microsoft Excel. [31] Outcomes will be grouped into key themes (groups of similar verbatim outcomes). A list of all unique outcome themes extracted will be created. The outcome themes will be classified as outlined by COMET taxonomy into domains. [15] The data will be written up in accordance with guidelines from PRISMA (Preferred Reporting Items for Systematic Reviews and Meta-Analyses). [38]

#### Output

A list of adverse outcomes reported in the literature and how they are measured. This list will be used in conjunction with the outcomes from WP2 in WP3.

### WP2—Qualitative patient interviews to generate a list of adverse outcomes important to patients and carers

WP2 will consist of semi-structured qualitative interviews to ensure that any key outcomes of importance to patients and their carers are identified. Paediatric COS in other specialities have utilised surveys to define the outcomes of significance to patients and their carers. [39] Given the sensitive nature of discussing adverse outcomes following surgery for a devastating diagnosis such as a paediatric brain tumour, the use of an interview format will allow discussion and the opportunity to ensure the participant is well supported.

#### Research question

What adverse outcomes do paediatric neuro-oncology patients and their carers feel were the most important following a surgical procedure?

#### Types of participants

Patients who are children over 8 years of age or now adults and their carers are eligible to participate. Traditional semi-structured interviews in children under 8 years old are thought to not yield meaningful information. [40] Interviews will be restricted to those 8 years old or above at the time of interview. Patients with varying levels of neurological disability will be recruited to ensure broad representation. Adults who are unable to consent and individuals who cannot speak English will be excluded due to the likelihood of difficulties engaging with the process. Paediatric patients need to be able to engage with the process and give assent for participation.

Recruitment will be through outpatient clinics and local databases at Alder Hey Children’s Hospital. The databases include basic demographic data as well as information about the pathology and surgical intervention undergone by the patients. Stratified purposive sampling and matrix sampling will be used to ensure there is fair representation of the different tumour types and locations (Table 3). We will initially seek to involve patients or their carers more than 6 months post procedure and less than 5 years post procedure. We will make a list of all eligible patients and carers from the database and will use randomised sampling to choose which to contact. Patients and carers will not have to be matched. Diagnosis of a cranial brain tumour will be radiologically and histologically confirmed.

**Table 3:** Sampling matrix for qualitative interviews

Tumour location	Patient	Parent/carer
Pituitary/suprasellar/optic pathway/hypothalamic glioma/craniopharyngioma/anterior third ventricle	2	2
Thalamic/pineal/tectal plate/midline supratentorial/posterior third ventricle	2	2
Supratentorial hemispheric/lateral ventricle	2	2
Posterior fossa/fourth ventricle—medulloblastoma	2	2
Posterior fossa/cerebellopontine angle—ependymoma	2	2
Posterior fossa/cerebellar hemisphere/brainstem—astrocytoma	2	2

The interview will be initially trialled with the patient and carer research partners in the SAG. One patient and one carer will be interviewed as a pilot to ensure our interview technique and structure is fit for purpose, with adjustments made if needed. Following this, the remaining patients and carers will be interviewed until data saturation is attained.

One study reports that code saturation can be achieved in 9 interviews. [41] Given the variations in disease type, location and patient/carer role reflected in our sampling matrix, we anticipate we will need to interview 24 individuals to achieve data saturation but acknowledge the number for this may be as high as 40. [42] If data saturation has not been achieved after 24 patients, then 1 more patient and carer will be interviewed in that group until it has been achieved.

Guidelines for assessing saturation in qualitative research remain vague with limited evidence base. [41, 43] The interviews aim to identify outcomes of importance to patients and their families that have not been highlighted by the systematic review, for inclusion in the consensus process. Code saturation, defined as the point where no additional themes are identified, will be used, with data saturation being defined as no new outcomes identified for the last 2 participants of interviews for each group. This will be in comparison to the themes generated from the interviews of each group, as well as the themes already identified from the systematic review.

#### Types of interventions

Paediatric patients who have undergone an intracranial neurosurgical intervention and their carers will be eligible for this study. Full inclusion and exclusion criteria can be found in Table 4.

**Table 4:** Inclusion and exclusion criteria for qualitative patient interviews and consensus process

Inclusion criteria	Exclusion criteria
• Participants need to be 8 years old or above at time of interview • Paediatric patients (children and young people) with a brain tumour up to and including the age of 18 years at the time of diagnosis and their parents or carers • All brain tumours—benign and malignant • Definitive surgical interventions including resection and biopsy	• Participants under the age of 8 years at the time of interview • Adult patients over the age of 18 at diagnosis and their carers • Spinal tumours • Non-neuro-oncological pathologies • Isolated surgical adjuncts such as external ventricular drains, endoscopic third ventriculostomy or ventriculoperitoneal shunts • Isolated non-surgical interventions such as such as pharmacological therapy, chemotherapy, radiotherapy, radiosurgery, peri operative measures and supportive treatments or therapies • Adults unable to consent for participation • Minors unable to assent for participation • Non-English speakers

#### Recruitment and registration

Eligible patients and carers will be contacted through details stored on their medical records, accessed by the lead investigator (STK) or members of the project supervisory team who are part of the clinical care team and have permissions to access this data (CM, BP). Those identified in outpatient clinics will be given an invitation letter and information sheets directly face to face and will be signposted to the expression of interest form to indicate if they want to take part.

Participants will be contacted with an invitation email from the clinical team accompanied by an information sheet. The information sheet will have a link to an expression of interest form where participants can indicate 3 options—‘I/my child would like to take part in the interview/survey’, ‘I would like to be contacted with more information about the study’ or a ‘I/my child DO NOT want to take part in the interview/survey’ option. There will be multiple information sheets available according to the role and age of the participant. There will be a 3-week period to opt into participating in the project, with a clear date noted in the invitation letter and the information sheet. This form will be on the University of Liverpool’s JISC (Joint Information Systems Committee) surveys [44] account and the results will be sent directly to a secure NHS email address. In addition to completing the JISC form, [44] there will be a secure NHS email address to reply to if they do not want to participate. After 3 weeks, if no response has been received then we will contact the potential participant to address any questions or concerns and confirm participation.

A recruitment survey will be administered online, which will include details such as demographics, ethnicity, medical and oncology background and contact details. A unique identifier will be allocated to each participant to ensure anonymity. Participants will be offered renumeration vouchers for the value of £25 for participating and can choose between vouchers for Love to shop, several supermarkets or Amazon.

#### Consent

Written consent will be obtained electronically. A meeting between the lead investigator (STK) and each participant will take place to answer any questions, sign the consent and assent forms and set a date and time for the interview. Consent will be recorded electronically through the JISC surveys system [44] and confirmed at the start of the interview.

Consent will be obtained from the person with legal parental responsibility for the child involved. Written assent will be obtained from patients who are minors for participation in the project.

A patient’s right to dissent to participate will be respected and participants will be free to withdraw from the study at any point. They will be able to withdraw their interview up to 1 week after their interview. After this time, data collected up to withdrawal will be used due to difficulty separating from other data. If participants are not able to complete the interview due to distress or fatigue, then they will still receive the renumeration voucher. This will be made clear during the consenting process.

#### Interview format and analysis

Semi-structured qualitative interviews by the lead investigator (STK) will be used to encourage participants to discuss their lived experiences. Methods will be adjusted according to the age and role of the participant. Online interviews through a university or NHS Microsoft Teams [45] account will be offered in the first instance, to ensure compliance with data protection guidelines. The interviews will last 45 min to 1 h, with the option of ending the interview early if the participant is feeling fatigued. Children will be able to choose if they do the interview with their parent or guardian. Family units will be involved as a whole if this is the case. Interviews will be audio and video recorded and the software will be used to generate transcriptions, which will be checked against the recording. The transcriptions will be imported into NVivo [32] and analysed, extracting the outcomes. A list of all outcomes, both favourable and unwanted, will be compiled from the transcriptions for transparency. The structure and questions for the interview will be reviewed ahead of time by the SAG members with transcripts reviewed by an independent validator to ensure consistency in conduction of the interview.

The qualitative data extracted will be analysed using a thematic framework analysis. [46] This allows for the identification of patterns and grouping of outcomes into themes. [47] Grouping of terms and phrases into key themes will be clearly recorded and discussed with the SAG to ensure reliability. Themes identified will be incorporated into the COMET taxonomy [48] used in the systematic review, allowing for ease of combining and deduplicating the two lists. Results will be reported in line with the COREQ (Consolidated criteria for reporting qualitative research) checklist. [49]

#### Outputs

A list of adverse outcomes of importance to patients and carers and information to support plain language outcomes for use in WP3.

### WP3—Consolidation of outcomes and seeking consensus on critically important adverse outcomes (eDelphi survey and consensus meeting)

WP3 will consolidate outcomes for the consensus process. Critically important outcomes will be identified through an eDelphi survey, with ratification of the final COS at a half day consensus meeting.

#### eDelphi survey

Outcomes from WP1 and WP2 will be consolidated with appropriate lay descriptions. The final list will be reviewed the SAG to ensure the grouping and lay description of each outcome is appropriate.

##### Research question

What outcomes do key stakeholders groups including patients, carers, healthcare professionals and researchers believe should be included in a COS for use in reporting morbidity following surgery in paediatric neuro-oncology patients?

##### Participants

Three key stakeholder groups of (1) patients and carers, (2) healthcare professionals and (3) researchers will be invited to take part in the eDelphi survey. Inclusion and exclusion criteria are outlined in Table 5.

**Table 5:** Inclusion and exclusion criteria for consensus process

Inclusion criteria	Exclusion criteria
• Patients over the age of 8 years who have undergone a surgical procedure for a brain tumour up to and including the age of 18 years at the time of diagnosis • Parents and carers of patients who have undergone a surgical procedure for a brain tumour up to and including the age of 18 years at the time of diagnosis • Healthcare professionals and researchers involved in the care of postoperative paediatric brain tumour patients	• Adult patients over the age of 18 years at diagnosis • Spinal tumours • Non-neuro-oncology pathologies • Patients with isolated adjuvant surgical interventions such as external ventricular drains, endoscopic third ventriculostomy or ventriculoperitoneal shunts

Involvement of children in the Delphi process is rare with few published studies and no standard guidelines. Current standards such as COS-STAD [23] and the COMET taxonomy [48] were initially developed for use in adult populations, with calls to include items applicable to children as well. [50] The development of previous paediatric COS has been centred around consensus methods involving healthcare professionals and parents/carers rather than children and young people. Only 31% of paediatric COS registered with COMET in 2022 involved children and young people in at least one part of the study, with only 25% involving them in outcome listing and consensus. [30] Several studies have described difficulty with recruitment and retention of children and young adults. This is due to utilising the same methods as adults. [51] Measures such as animations instead of written information and clear communication about the importance of core outcome sets, including a patient perspective, were identified by a workshop of children and young people as being key in addressing this problem. [51] Some studies have restricted involvement of children and young people to those of older ages [52] whilst others have used adapted scoring systems such a modified Wong-Baker scales [25] or traffic light systems [26] with a corresponding Likert style scale to improve engagement. Feedback from a workshop of children and young people advised that a 9-point Likert style scale was too complicated. Alternative formats such as traffic light system or descriptive terms used for the GRADE scale such as ‘not important’, ‘important’ and ‘very important’ were deemed more suitable. [51]

The methodology for eDelphi survey for children will be guided by the SAG and will incorporate the use of modified scales such as the Wong-Baker scale [25] or a traffic light system. [26] The patients and carers in the SAG will review proposed methods and we will carry out a pilot trial with a small group of patients and carers with a think aloud process to ensure the scoring systems is appropriate and fit for use.

Healthcare professionals and researchers will be recruited through scientific conferences and through professional societies such as the European Society for Paediatric Oncology (SIOP-E), the Society of British Neurological Surgeons (SBNS) and the British Paediatric Neurosurgical Group (BPNG). This project has been presented internationally and has generated wide interest from healthcare professionals in multiple international centres for involvement in the eDelphi survey, including centres in USA, Central and South America, India and Africa as well as centres in the UK and Europe. Members of the SAG and key collaborators will be encouraged to distribute information for recruitment at their institutions. Guidance will be sought from the SAG about key international societies to contact to ensure balanced representation from all clinical specialities and multidisciplinary roles. Authors of the published and ongoing studies included in the systematic review will be contacted and invited to participate.

##### Sample size

There is no standard method for sample size calculation for the Delphi process. [26] Literature has shown that group error is reduced with increased group size. [53] Replicability of rated items has been shown to be around 80% with a sample size of 60–80, with this increasing to 83% with a sample size of 80–160. A sample of size of 20–30 per key stakeholder group may be sufficient. [54] A large patient and carer population would ensure that different pathologies are included and minimise any bias. This project aims to involve 100 healthcare professionals and researchers and 100 patients and carers, assuming a 20% attrition rate with a final sample size of 80 healthcare professionals/researchers and 80 patients/carers. There is no minimum sample size for involvement in the consensus process and so if after the initial recruitment phase our numbers are lower, we will discuss this within the SMG. If the numbers are deemed acceptable, we will proceed with the consensus process.

##### Recruitment and registration

Healthcare professionals and researchers will be contacted by email in the first instance, whilst patients will be contacted via patient charities and through outpatient clinics and local databases described previously at Alder Hey Children’s Hospital. An invitation letter and information sheets about the project will be distributed via email which will include a link to the study website and a link to the expression of interest form.

Healthcare professionals and researchers will be contacted by email in the first instance, whilst patients will be contacted via patient charities and through outpatient clinics and local databases described previously at Alder Hey Children’s Hospital. An invitation letter and information sheets about the project will be distributed via email which will include a link to the study website and a link to the expression of interest form.

The Brain Tumour Charity [28] and Thumbs Up For Charlie Charity [29] will aid with recruitment for the consensus process. The Brain Tumour Charity [28] will facilitate the distribution of posters in outpatient clinics and wards in hospitals across the UK with a link to the study website. They will also distribute information on social media and at family days out for patients and their carers to express interest to participate in the consensus process.

Participants will be asked to complete the expression of interest form which can be accessed through the website or information sheets. At the end of the registration period, those who have requested a phone call will be contacted to address any questions they may have. If there are too few individuals registered for participation at the end of the registration period, then potential participants who have not replied will be contacted to address any further questions or concerns, or difficulties engaging with the process. This will only be possible for the patients who have been identified from the outpatient clinics and databases at Alder Hey Children’s Hospital as the contact details for those recruited from the charities will not be available.

Participants will be asked to complete a recruitment survey at the start of the first round of the Delphi survey. At registration, basic demographic information will be recorded. They will be asked to confirm which stakeholder group they belong to. Baseline demographics such as age, gender, ethnicity and geographical location will be recorded for all participants. For healthcare professionals, job role, years of experience and country of clinical practice will be recorded.

For patients, the type and location of the tumour and surgical procedure will be recorded as well as details of whether they have completed the survey themselves or if a parent/carer has helped. Age at diagnosis will be recorded as well as time since diagnosis. The same information will be recorded for carers, as well as information about their specific role and the time scale during which they were caring for the patient. All data will be anonymised and will be stored as described above.

We will also record information about the recruitment process for all participants including how they have heard about the project and key motivators for taking part. Given the sparsity of literature on the involvement of paediatric patients in the Delphi process, it will allow insight into how to improve recruitment for future research.

We will not offer renumeration for participants of the Delphi survey or consensus meeting. This is in line with standard practice for this type of research. Discussion in a workshop with children and young people also identified issues with offering financial incentives as it may encourage some people to register and not complete participation or participating without thinking of their responses. It was suggested that certificates of participation might be more useful in encouraging children and young people to participate. [51]

##### Consent

Participants in the eDelphi will provide electronic consent prior to accessing the survey. For participants under 16 years of age, electronic assent will be sought alongside electronic consent for the person with parental responsibility.

##### Questionnaires

Participants will rate outcomes in a two-round eDelphi survey. The survey and instructions for completion will be piloted and reviewed with the members of the SAG.

The eDelphi survey will be distributed for completion to registered participants. Instructions on how to complete the survey and contact details for any questions or concerns will be distributed with the link for the survey. Participants will be asked to complete the survey within a 4-week period, with reminders at 2 weeks, 1 week and 48 h prior to the close of the survey. Failure to complete the first round of the survey will mean exclusion from the second round and consensus meeting.

Outcomes will be grouped by themes as per the COMET taxonomy. Both plain language terms and scientific definitions will be included. Additional outcomes can be suggested by participants at the end of round one. These will be reviewed by the SAG prior to inclusion in round two. Following completion of round one, the ratings will be summarised and the grouped results, by stakeholder group, shared with all participants in round two. Round two will take place over a period of 4 weeks, with reminders to complete the survey as per round one.

The survey will be completed after two rounds whether consensus has been achieved or not. The final summary of the ratings will be prepared for the consensus meeting.

##### Rating

Participants will be asked to rate the outcomes using a 1–9 Likert style scale (1 not that important, 9 critically important) or corresponding alternative scale for younger patients to use. Both adults and children will complete the two rounds of the survey. Table 6 details the definition of consensus for inclusion of outcomes in the COS.

**Table 6:** Definition of consensus for inclusion of outcomes in COS

Consensus classification	Description	Definition
Consensus in	Consensus that outcome should be included in the COS	80% or more participants, in each stakeholder group, scoring as 7–9 (or equivalent on the corresponding paediatric scoring system) and < 15% participants, in each stakeholder group, scoring as 1–3
Consensus out	Consensus that outcome should not be included in the COS	50% or fewer participants scoring 7–9 (or equivalent on the corresponding paediatric scoring system) in each stakeholder group
No consensus	Uncertainty about importance of outcome	Anything else

##### Attrition bias

Attrition between rounds can be a potential source of bias in this project. There is no clear guide as to what is an acceptable response rate but 80% is quoted in most instances. [15] This project aims for an attrition rate of 20% or less. Attrition bias will be assessed between each round of the survey. The effect of attrition in round two will be assessed by calculating the average ratings for the outcomes from round one and comparing it those completing both rounds. [55]

Regular engagement with participants through email and information on the website will be utilised to reduce attrition. Information provided on the patient information sheets will emphasise the importance of completing both surveys. Participants will receive regular personalised email reminders to try to reduce attrition. If the attrition is more than 20%, we will extend the time available to complete the round.

##### Output

A list of rated outcomes grouped by the consensus classifications described in the definition of consensus.

#### Consensus meeting

Following completion of the eDelphi survey, an online half day consensus meeting will be held to ratify the final COS.

##### Research question

Can consensus be achieved, and a COS be ratified from outcomes rated by key stakeholder groups in the eDelphi survey?

##### Participants

An equal representation of all key stakeholder groups will be invited to participate, with up to 30 participants in total. This includes 10 patients and carers, 10 healthcare professionals and 10 researchers who have completed both rounds of the eDelphi survey. Stratified purposive sampling will be used to ensure a diverse representation of participants.

##### Consent

Participants will be able to indicate if they would like to take part in the consensus meeting by answering a question in round two of the survey. Consent will be implied by attending the consensus meeting.

##### Method

An independent facilitator will run the meeting. Outcomes with clear consensus, whether that is for inclusion or exclusion, will be reviewed with limited discussion. Outcomes with no consensus will be prioritised before the meeting and discussed by participants with subsequent rating during the meeting. The same criteria for consensus as applied in the eDelphi survey will be used in the consensus meeting (Table 6). Outcomes that reach ‘consensus in’ will be included in the final COS.

##### Outcome

Final COS of which adverse outcomes should be reported following surgery in children with brain tumours ready for dissemination and uptake by the international community. This will inform further research into how, when and by whom each outcome should optimally be measured.

### End of study

The end of study will be classified as the final point at which data will be collected for the core outcome set. In this project, this will be after the consensus process once the outcomes for inclusion have been finalised.

### Potential barriers

This project will create a core adverse outcome set for use in paediatric patients undergoing surgery for a brain tumour, to standardise reporting of morbidity internationally, in both research and clinical services. To achieve this, the COS needs to be applicable to patients across the world. The involvement of multiple centres internationally in the consensus process will ensure that regional variations in care delivery and cultural perceptions of morbidity are taken into account. The opportunity to suggest outcomes for inclusion in the second part of the Delphi survey provides an opportunity for international participants to suggest any outcomes that would be of importance to their communities. This could include outcomes that may differ due to variations in culture or religion, or those that may be considered important due to the availability of resources in some settings. The involvement of key international scientific communities will allow the opportunity to reach healthcare professionals and researchers in more remote settings, through the distribution of invitations to participate through mailing lists.

### Outputs and dissemination

There is a recognised issue with the reporting of postoperative morbidity in paediatric neuro-oncology. The recent GIRFT report has highlighted the need for outcome measures with inclusion of patient-reported outcomes, long-term outcomes and impact on the family. This project will address this by producing a COS for these patients that will highlight the morbidity and life impact associated with surgical interventions.

This COS will ensure that adverse outcomes recorded to assess surgical success are relevant to the patient population. The GIRFT report has highlighted the importance of including patients views in the generation of this COS. Patients and carers will be involved at all stages of the project, including study development in the SAG and to assessing adverse outcomes of significance to them. This will highlight which PROs are critically important so measurement tools can be chosen carefully reducing the burden to healthcare professionals and patients and addressing the issue of time constraints.

Good uptake of this COS will ensure the impact of standardisation of reporting of morbidity nationally and internationally. The key to this is dissemination. Our findings from each stage of the project will be published in open access journals and disseminate findings at national and international conferences. A study summary will be shared with participants and patient organisations. The strong research network which has been developed around this project will encourage uptake. Endorsement from SIOP-E supports the use of this COS in prospective surgical research and clinical studies. Key recommendations from GIRFT and support of SBNS and BPNG will encourage uptake in the UK. The involvement and personal investment of multiple international centres in the development of the project and consensus process will encourage good uptake at an international level.

The final COS will be disseminated widely to ensure uptake. As well as the potential for application directly after publication, we anticipate there will be a delayed effect as it is incorporated into prospective research as standard. The encouragement from funding organisations such as the National Institute for Health and Care Research (NIHR) to include existing COS when planning prospective research with direction to search the COMET database [56] will encourage the use of this COS in prospective research studies. This will be further strengthened by the support from societies such as SIOP-E, SBNS and BPNG and the recommendation from GIRFT that as well as informing audits, consensus COS should provide a basis for future clinical trials in paediatric neuro-oncology surgery. [57]

This project will have a range of direct and indirect benefits for surgical paediatric neuro-oncology patients and their families including improvements in population health and improving quality of life by acknowledging and assessing morbidity. Accurate reporting of morbidity will enable clinicians, patients and carers to understand quality of services and treatments and will allow for quality improvement through service evaluation. [57] It will enable accurate documentation and comparison of morbidity at local, national and international levels and aid establishment of a benchmark for comparison, and can be applied to current clinical service evaluation following publication. Morbidity associated with current and new treatments can be assessed and consistent data collection in trials and clinical services will be encouraged. It will reduce research waste and lay the groundwork for further research in morbidity reporting, including standardising the tools used to measure each outcome.

## Study status

Protocol version 6, 28/6/2025.

Recruitment for WP2 and WP3 to start 1/5/2025 and complete 31/12/2025.

## Supplementary Information

Additional file 1: COS-STAP (Core Outcome Set-STAndardised Protocol Items) statement.

## Data Availability

N/A.

## Abbreviations

BPNG: British Paediatric Neurosurgical Group
CNS: Central nervous system
COMET: Core Outcome Measures in Effectiveness Trials
COREQ: Consolidated criteria for reporting qualitative research
COS: Core outcome set
COS-STAD: Core Outcome Set-Standards for Development
COS-STAP: Core Outcome Set-STAndardised Protocol Items
GIRFT: Get It Right First Time Programme
HRA: Health Research Authority
JISC: Joint Information Systems Committee
NHS: National Health Service
NIHR: National Institute for Health and Care Research
OMERACT: Outcome Measures in Rheumatology
PoPPIE: People and Patient Participation, Involvement and Engagement
PRISMA: Preferred Reporting Items for Systematic Reviews and Meta-Analyses
PRO: Patient-reported outcome
PROMs: Patient-reported outcome measures
REC: Research Ethic Committee
SAG: Study Advisory Group
SBNS: Society of British Neurological Surgeons
SIOP-E: European Society for Paediatric Oncology
SMG: Study Management Group
WP1: Work package 1
WP2: Work package 2
WP3: Work package 3
