# Supplementary material for: The COMBAT Project: study protocol for the development of a core outcome set for morbidity following surgery in paediatric brain tumour patients
Source: Trials. 2025 Aug 11;26:286. doi: 10.1186/s13063-025-09004-4 (PMC12341105; doi:10.1186/s13063-025-09004-4)
Supplement: Supplementary file 1 — Additional file 1: COS-STAP (Core Outcome Set-STAndardised Protocol Items) statement. [file 13063_2025_9004_MOESM1_ESM.docx]

**Additional file 1 - COS-STAP (Core Outcome Set-STAndardised Protocol Items) statement**

| **COS-STAP Item** | | | **Page number** |
| --- | --- | --- | --- |
| TITLE/ABSTRACT | | | |
| Title | 1a | Identify in the title that the paper describes the protocol for the planned development of a COS | 1 |
| Abstract | 1b | Provide a structured abstract | 4 |
| INTRODUCTION | | | |
| Background and objectives | 2a | Describe the background and explain the rationale for developing the COS, and identify the reasons why a COS is needed and the potential barriers to its implementation | 5, 6 |
|  | 2b | Describe the specific objectives with reference to developing a COS | 6, 7 |
| Scope | 3a | Describe the health condition(s) and population(s) that will be covered by the COS | 6, 10, 11 |
|  | 3b | Describe the intervention(s) that will be covered by the COS | 6, 10, 11 |
|  | 3c | Describe the context of use for which the COS is to be applied | 6, 7, 21, 22 |
| METHODS | | | |
| Stakeholders | 4 | Describe the stakeholder groups to be involved in the COS development process, the nature of and rationale for their involvement and also how the individuals will be identified; this should cover involvement both as members of the research team and as participants in the study | 6,7, 8, 10, 11, 14, 15, 17, 18, 21 |
| Information sources | 5a | Describe the information sources that will be used to identify the list of outcomes. Outline the methods or reference other protocols/papers | 7, 9, 11-21 |
|  | 5b | Describe how outcomes may be dropped/combined, with reasons | 13, 16, 20 |
| Consensus process | 6 | Describe the plans for how the consensus process will be undertaken | 17-21 |
| Consensus definition | 7a | Describe the consensus definition | 20 |
|  | 7b | Describe the procedure for determining how outcomes will be added/combined/dropped from consideration during the consensus process | 20 |
| ANALYSIS | | | |
| Outcome scoring/feedback | 8 | Describe how outcomes will be scored and summarised, describe how participants will receive feedback during the consensus process | 20 |
| Missing data | 9 | Describe how missing data will be handled during the consensus process | 20 |
| ETHICS and DISSEMINATION | | | |
| Ethics approval/informed consent | 10 | Describe any plans for obtaining research ethics committee/institutional review board approval in relation to the consensus process and describe how informed consent will be obtained (if relevant) | 8 |
| Dissemination | 11 | Describe any plans to communicate the results to study participants and COS users, inclusive of methods and timing of dissemination | 20, 22 |
| ADMINISTRATIVE INFORMATION | | | |
| Funders | 12 | Describe sources of funding, role of funders | 3 |
| Conflicts of interest | 13 | Describe any potential conflicts of interest within the study team and how they will be managed | 3 |
